# Fisheries dataset on moulting patterns and shell quality of American lobsters *H. americanus* in Atlantic Canada

**DOI:** 10.1038/s41597-022-01503-2

**Published:** 2022-07-07

**Authors:** Svenja Koepper, Shannon Scott-Tibbetts, Jean Lavallée, Crawford W. Revie, Krishna K. Thakur

**Affiliations:** 1 Department of Health Management, Atlantic Veterinary College, University of Prince Edward Island, Charlottetown, C1A 4P3 PE Canada; 2 Fishermen and Scientists Research Society, Halifax, B3M 4H4 NS Canada; 3 Canadian Council on Animal Care, Ottawa, K2P 2R3 ON Canada; 4 Department of Computer and Information Sciences, University of Strathclyde, Glasgow, G1 1XQ UK

**Keywords:** Marine biology, Population dynamics, Animal physiology

## Abstract

Monitoring the moulting phenology of American lobsters (*Homarus americanus*) is important for maintaining sustainable lobster stocks. Changes in lobster landings can affect reproduction and disease susceptibility. Data on lobster moult indicators and on life-history traits (sex, size) were collated from a twelve-year monitoring program (2004–2015) in six lobster fishing areas in Atlantic Canada. A total of 141,659 lobsters were sampled over 1,195 sampling events using a standardized protocol and commercial lobster fishing traps. The dataset contains pleopod stages, estimated hemolymph protein levels (°Brix values) and shell hardness as well as lobster sex and size. Evaluation of sex ratio dynamics is also possible but existing biases in sampling males and females needs to be noted. This dataset is valuable in terms of inferring spatio-temporal trends in the life history of lobsters, as well as in the analysis of their moult cycle, and hence more generally for fisheries science and marine ecology.

## Abstract (summary)

**Table:** 

Measurement(s)	Lobster sex • Lobster size • Lobster moult stage • °Brix value of hemolymph • Lobster shell hardness
Technology Type(s)	Eye • Vernier Caliper • Microscopic assessemnt of pleopods • Refractometer • Physical assessment of lobster carapace
Factor Type(s)	Year • Month • Lobster fishing area (LFA) • Location • Sampling event • Water depth
Sample Characteristic - Organism	Homarus americanus
Sample Characteristic - Environment	marine water body
Sample Characteristic - Location	Northwest Atlantic Ocean coastal waters of Canada

## Background & Summary

Increased landings of soft-shelled lobsters in southwestern Nova Scotia in the last 20 years has been a concern to the Canadian lobster industry^1,2^. Soft-shelled lobsters are considered lower quality because they have a lower meat yield and fare poorly during transportation and holding. American lobsters are the most valuable fisheries product in Atlantic Canada, generating $1.6B CAD out of the total of $3.2B CAD for all fisheries landings in this region in 2019^3^. Consequently, monitoring lobster shell quality and population dynamics is of great economic importance for local fisheries.

The range of the American lobster, *Homarus Americanus*, extends from North Carolina (USA) to the coasts of southern Newfoundland and Labrador (Canada)^4^. Lobsters moult repeatedly during their life cycle^4^. Moulting is divided into four main stages: intermoult, the time between two moults; premoult, where the new shell is formed and and body size swells temporarily through water absorption; the actual moult, when the old shell is repelled; and the postmoult period, when the new shell calcifies^5^. The premoult stage is further divided into ten substages inferred from changes in the pleopod appendages^6^. In the first year of growth, individual lobsters may moult up to ten times, and as size increases, moulting frequency decreases (Cobb 1976). Large individuals may only moult every other year^4^. Males tend to moult earlier than females in the breeding season (Cowan and Atema 1990) and female lobsters usually mate after moulting when their shell is still soft (Atema *et al*. 1979). Females do not usually moult while carrying eggs^7^.

As for all ectotherms, temperature affects most physiological processes in lobsters, including moulting^2^. Previous studies have reported that warmer temperatures lead to an earlier spring moult in lobsters in the Gulf of Maine (USA)^8^. A change in the timing of peak moulting could therefore change the proportion of postmoult lobsters in fisheries catches. These still have a soft shell and are vulnerable to intraspecific competition. In this stage, new tissue slowly replaces the water that was taken up during postmoult^9^. Accordingly, soft-shelled lobsters have lower protein levels and meat yields compared to intermoult lobsters which can have commercial implications for the fishery^2^.

To monitor changes in the moult cycle and shell quality of American lobsters in Atlantic Canada, the Atlantic Veterinary College Lobster Science Centre (Charlottetown, PE) implemented the Atlantic Lobster Moult and Quality Project in 2004. The Fishermen and Scientists Research Society contributed to the regular lobster samplings in Nova Scotia until 2014^2^. Here we present data on moult stage, shell hardness, estimated hemolymph protein levels, lobster sex, and size that were collected by trap hauls during and outside of the commercial fishing season. The dataset includes changes in lobster characteristics over twelve years and over six different lobster fishing areas. Previous research on this dataset has already identified risk factors for soft-shelled lobsters in Atlantic Canada^1^.

## Methods

### Data collection

The present dataset was collected within the framework of the Atlantic Lobster Moult and Quality (ALMQ) project originally managed and implemented by the Atlantic Veterinary College Lobster Science Centre at the University of Prince Edward Island in collaboration with the Fishermen and Scientists Research Society. The Atlantic Lobster Moult and Quality project was initially funded through the Atlantic Innovation Fund program from the Atlantic Canada Opportunities Agency (ACOA) and transferred to the Fishermen and Scientists Research Society (FSRS) in 2012.

**Fig. 1:**
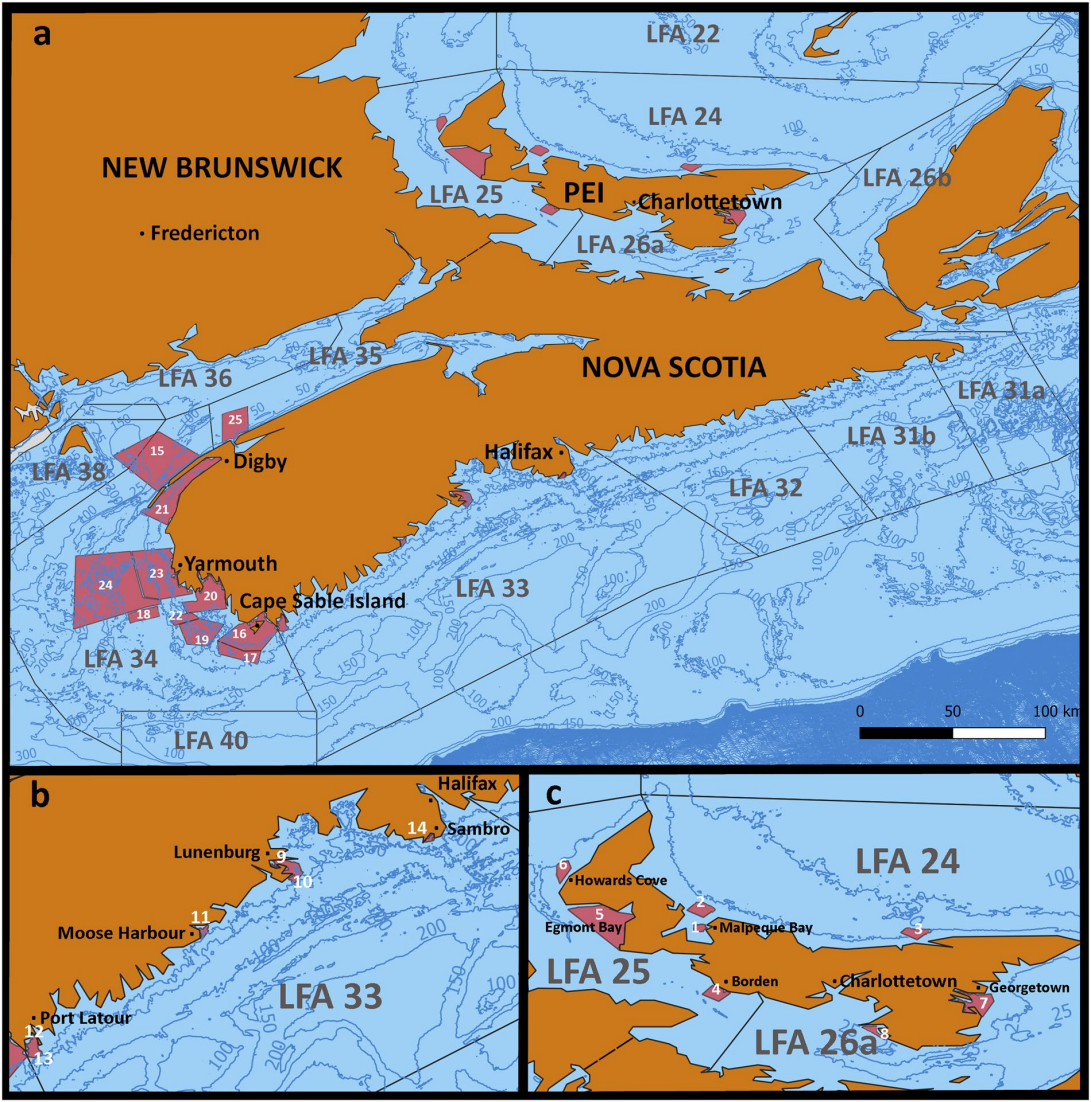
(**a**) Map of the lobster fishing areas (LFAs) in the Maritime Provinces in eastern Canada with the sampling locations (red) recorded by the AVC Lobster Science Centre for the Atlantic Lobster Moult and Quality project. (**b**) Enlarged map of LFA 33. (c) Enlarged map of LFAs on Prince Edward Island. The maps were created using QGIS (v. 3.18; https://qgis.org). Contours depict water depths in meters.

Sampling took place every 2–3 weeks in eight lobster fishing areas (LFA) in Atlantic Canada from 2004 to 2014 (see Fig. 1, Table 1). The sampling followed the FSRS Lobster Moult and Quality sampling protocol and was conducted by technicians from the Atlantic Veterinary College and the Fishermen and Scientists Research Society in fixed locations from traps set the day before^2^. Locations based on targeted sampling (LFA 33 and 34) were chosen according to the fishing efforts in the respective areas and selected by a lobster science committee consisting of members from industry, academia, research and federal and provincial representatives. Other locations (LFA 24, 25, 26A, 35) were chosen based on proximity to the Atlantic Veterinary College and other projects with commercial fishers which allowed sampling.

**Table 1:** Overview of sampling locations, surface areas (km^2^) and number of lobsters (N) sampled for the Atlantic Lobster Moult and Quality Project by AVC Lobster Science Centre from 2004–2015 in Atlantic Canada. (PEI = Prince Edward Island, NS = Nova Scotia).

Province	LFA	Area (sqkm)	Fishing season	Minimum legal size	Location	ID	Area (sqkm)	N
PEI	24	14,283	Apr. 30^th^–June 30^th^	73 mm	Malpeque Bay Inside	1	56	355
					Malpeque Bay Outside	2	192	100
					Morell	3	271	473
	25	5,830	Aug. 9^th^–Oct. 10^th^	77 mm	Borden	4	45	1,838
					Egmont Bay	5	271	2,070
					Howard’s Cove	6	88	3,113
	26A	7,308	Apr. 30^th^–June 30^th^	73 mm	Georgetown	7	194	3,875
					Pinette	8	27	1,445
NS	33	30,765	last Monday of Nov.–May 31st	82.5 mm	Lunenburg Inside	9	19	4,970
					Lunenburg Outside	10	107	862
					Moose Harbour	11	13	2,951
					Port Latour Inside	12	21	16,381
					Port Latour Outside	13	49	2,396
					Sambro	14	28	4,834
	34	25,538	last Monday of Nov.–May 31st	82.5 mm	Bay of Fundy	15	1,234	2,516
					Cape Sable Island Inside	16	311	6,300
					Cape Sable Island Outside	17	213	3,548
					German Bank	18	205	3,387
					Jaquard’s Ridge	19	398	16,341
					Lobster Bay	20	278	22,515
					St. Mary’s Bay	21	459	4,901
					Trinity Ledge	22	81	200
					Yarmouth Inside	23	917	20,540
					Yarmouth Outside	24	1,924	15,373
	35	5,432	Oct. 15^th^–Dec. 31^st^, Mar. 1^st^–July 31^st^	82.5 mm	Digby	25	489	375

For each sampling event, 40 commercial lobster traps with escape vents for lobsters below the minimum legal size were used. Legal sizes depend on size-at-maturity (size at which 50% of the population reach maturity) which differs between LFAs due to regional differences in water temperature that influence lobster growth. There were some differences in sampling procedure between lobster fishing season and off-season. During lobster fishing season sampling took place within 48 h post landing and only legal-sized lobsters were assessed. During off season, lobsters were sampled directly on board chartered boats and were returned to sea immediately after sampling. During non-fishing season sampling, lobsters below minimum legal size were also sampled but no egg-bearing females were targeted to minimize negative handling effects. Targeted sample size was 200 lobsters per sampling event before 2009 and 125 lobsters after 2009 due to budget constraints.

**Fig. 2:**
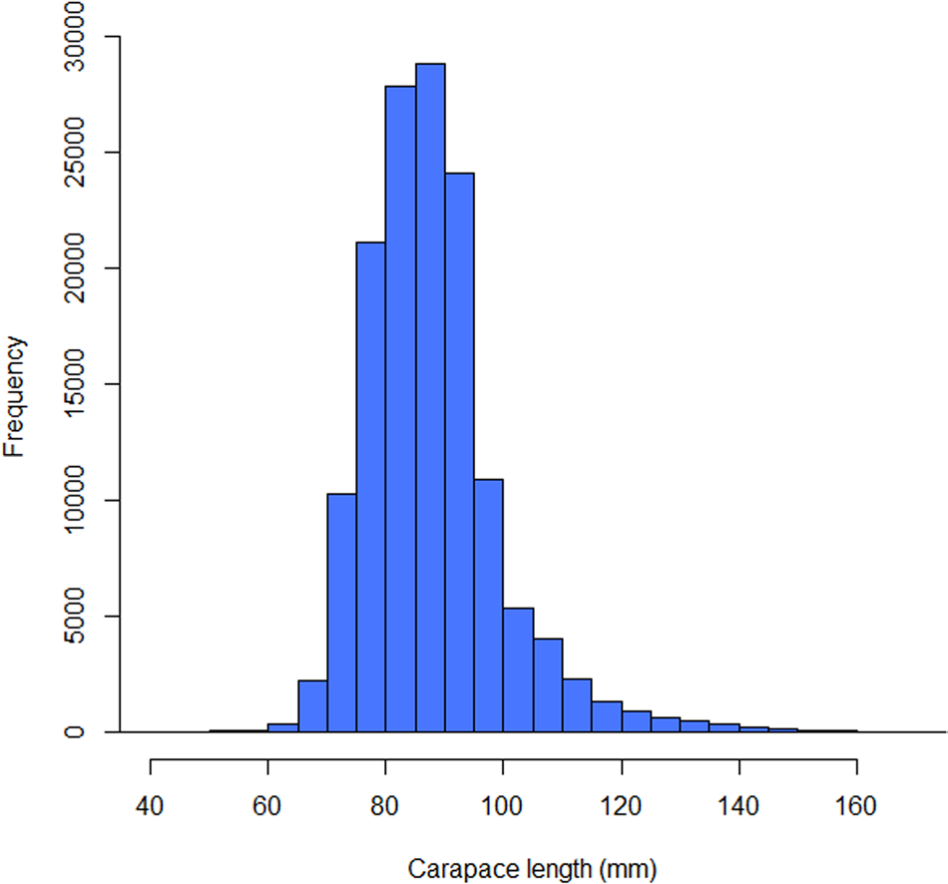
Lobster size (as carapace length in mm) distribution for all lobsters sampled during the sampling period (15 missing values).

On average, 3–4 lobsters of each sex were sampled in every 2 mm lobster size grouping. Lobster size was recorded as the carapace length in mm and determined using calipers rounding down to the nearest mm. The size distribution of sampled lobsters is presented in Fig. 2. Lobsters were assessed for general health (lesions, shell damage, liveliness/vigour) and shell hardness. Shell hardness was recorded as soft, medium or hard. A carapace of a soft-shelled lobster would be compressible at the ventral and dorsal (anterior and posterior) carapace, a medium-shelled lobster would only be compressible at the ventral carapace and a hard-shelled lobster would not be compressible at any carapace location.

**Fig. 3:**
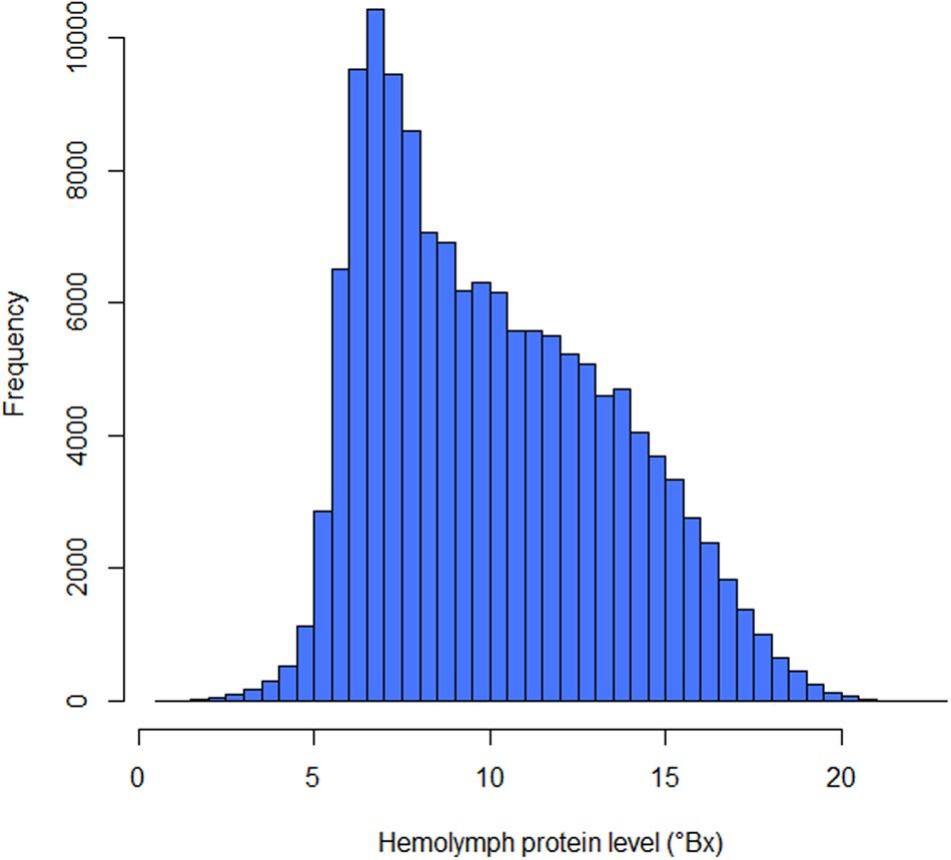
Distribution of hemolymph protein level (measured in °Brix) for all lobsters sampled in the dataset (892 missing values).

**Fig. 4:**
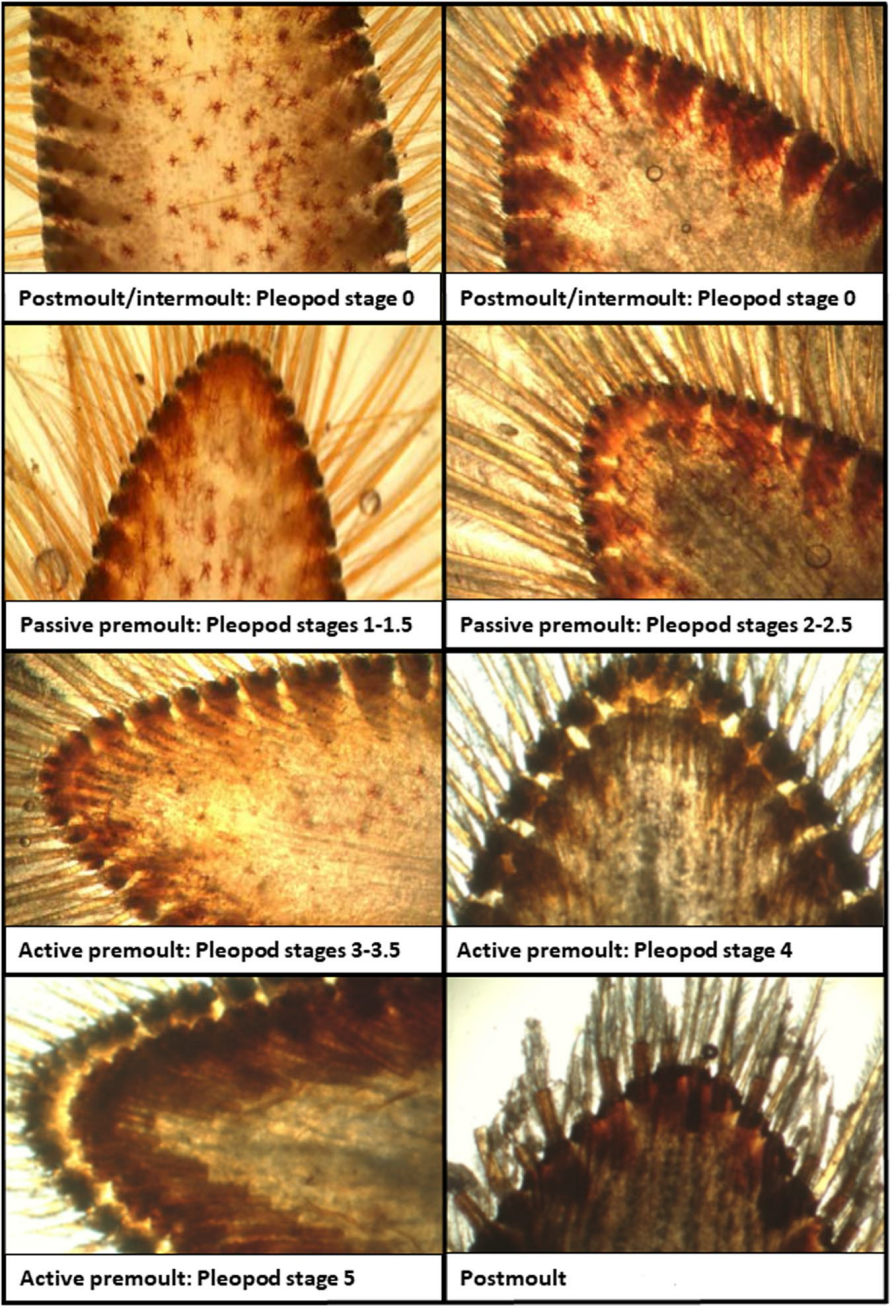
Pleopod stages of lobsters at different times in their moult cycle. Illustrations by Lavallée *et al*.^2^.

To estimate hemolymph protein levels, the ventral abdomen between the first pair of walking legs was sprayed with 70% ethanol and 3 ml of hemolymph were extracted with a 22 gauge needle and a 3 ml syringe. A few drops of hemolymph were placed on a handheld refractometer and the refractive index (“°Brix” value) was recorded and used as a proxy for total hemolymph levels. The distribution of hemolymph protein level is shown in Fig. 3. The moult stages were determined by pleopod stages under a stereomicroscope and recorded in pleopod stages (see Table 2). The stage determinations are shown in Table 2 and Fig. 4^6^.

**Table 2:** Description of premoult stages and pleopod stages in adult American lobster based on Aiken^6^. C: Intermoult, D: Premoult.

Molt stage	Pleopod stage	Description
C4	0	Epidermis closely applied to cuticularnodes at tip of pleopod; no amber zone or epidermal retraction at pleopod tip
D0'	1	First indication of apolysis - amber or double-bordered region forms at the pleopod tip; chromatophores often show signs of reorganization, but there is no epidermal retraction from the cuticle
D0″	1.5	Epidermis retracting from the terminal cuticular nodes; may have double-bordered appearance
D0″	2	Epidermal line clearly formed and retracting from lateral cuticular nodes
D0″′	2.5	Maximun epidermal retraction - not touching any lateral cuticular nodes
D1'	3	Invagination papillae form at site of future setae; epidermis becomes scalloped
D1″	3.5	Invagination papillae clearly formed, but shafts of new setae not well defined
D1″′	4	Shafts of developing setae visible but proximal ends not clearly defined; shafts now invaginated to maximum length
D2'	4.5	Shafts visible full length, but proximal ends are bifurcate instead of blunt; barbules becoming visible on setal shafts
D2″	5	Shafts of developing setae thick, proximal ends blunt
D3	5.5	Shafts of setae very thick and dark, proximal ends blunt; classify as D3” if fold or ripples are visible in cuticle on upper surface of pleopod

In total, 141,659 lobsters were sampled from 2004–2015 over 1,195 sampling events. Data were recorded manually on data sheets and re-checked before being entered into an Excel data sheet (Excel, Microsoft).

## Data Records

The data fields are described in Table 3. The data represent moult stage and shell quality of each sampled lobster together with additional data on lobster characteristics (sex, size) and spatial-temporal information over the 12-year sampling period. The table consists of 141,659 rows and 15 columns and each row represents one sampled lobster. To ensure uniqueness of the records, the associated information for each sampling event is reported as several lobsters were sampled per sampling event. Summary statistics for continuous and categorical variables are presented in Tables 4 and 5. Data are published on the data depository Zenodo (10.5281/zenodo.6588031)^10^.

**Table 3:** Description of the fields in the dataset.

Field	Description
SampleID	Unique Atlantic Veterinary College Lobster Science Centre sample ID
Date	Date on which sample was aquired (DD-MM-YYYY)
Month	Two-digit month of sampling
Year	Four-digit year of sampling
LFA	Lobster fishing area sampling location is in
Sampling event	Unique number associated with each sampling event
Location	Location where samples were fished
LocationID	Unique number associated with each of the sampled locations
Sex	Lobster sex
Size	Lobster carapace length (CL) in mm
Min_legal_size	Minimum carapace length for lobsters to be legally landed
Season	Sampled during or outside of the fishing season
Moltstage	Molt stage according to pleopod staging
Moltstage_Group	Molt stage group
Brix_st	°Brix value for hemolymph protein concentrations
Shellhardness	Shell hardness
Water_depth_m	Water depth in meters

**Table 4:** Descriptive summary of continuous variables. NA = missing values.

	N	NA	Min	Max	Mean	Median
Brix_st	140,767	892	0.8	22.8	10.13	9.6
Size	141,644	15	5	192	88.37	87
Water_depth_m	114,129	27,530	4.03	137.16	21.12	12.15

**Table 5:** Descriptive summary and frequencies of categorical variables. NA = missing values.

	N	NA	Frequencies										
Sex	141,639	20	Male (1)				Female (2)			Ovigerous female (3)			
			76,768				64,856			15			
Moltstage	141,485	174	0	1	1.5	2	2.5	3	3.5	4	4.5	5	5.5
			80,722	12,559	11,422	13,922	12,142	5,820	2,637	1,308	401	379	173
Moultstage group	141,485	174	Postmoult/ Intermolt (1)				Passive premoult (2)			Active Premoult (3)			
			80,722				50,045			10,718			
Shellhardness	141,659	—	Soft (1)				Medium (2)			Hard (3)			
			4,347				27,050			110,262			

## Technical Validation

A standardized sampling protocol was followed over the whole study period to avoid bias due to the fact that sampling was conducted by a variety of staff. In 2009, the sampling size changed from approximately 200 lobsters per sampling event to 125 due to budget constraints.

The dataset was validated by repeated data quality checks at time of sampling and sample processing. In the former, errors were corrected where appropriate. In the latter, diagnostic plots and tables were checked and outliers were identified by the 1.5 interquartile rule (Figs. 2, 3). If outliers were out of the biological range, they were then treated as data entry errors and classified as missing values (“NA”). Location data were validated by checking the sampling locations in Google Maps to ensure correct matching with the associated LFA data.

## Usage Notes

With more than 140,000 observations, our dataset provides valuable information on moult indicators and other lobster characteristics in regions that are of high commercial value for lobster fisheries. It is important to note that the trap-based design of the survey may not represent the true natural lobster population unless an approach to adjust for sampling bias is accounted for. For example, lobsters that are close to or have just moulted are shelter seeking and would likely not encounter traps. It was not feasible to sample all locations consistently at the same time resulting in some locations being less represented than others. This could be a limiting factor for future analyses.

Data transformation and Gaussian curve fitting can be implemented to estimate peak moulting time and compare it across years and within locations. While the estimation of hemolymph protein levels and pleopod staging are objective, the shell hardness assessment is subjective methods as it was carried out by manual squeezing of the lobster carapace. This should be considered when using these variables as moult indicators during analysis.

Caution should be taken when analysing sex ratio patterns with these data as the sampling protocol targeted an equal number of males and females per sampling. However, a 50:50 sex ratio was recorded in only 21 of the 1,195 sampling events. This indicates that trends in sex ratio patterns could be inferred from this dataset. The fact that either not enough males or females were present in the traps to reach equal sampling frequency could indicate skewed sex ratios in the lobster population, temporal shelter-restricted behaviour differences between gender, or reluctance for one gender to enter a trap if larger lobsters of the other gender are already present. This could be further analysed by mixed effect linear regression models.

A limitation of this dataset is that temperature data are missing for sampling events and thus for the individual observations. It is possible to obtain satellite based retrospective sea surface temperature data from databases such as NOAA (National Oceanic and Atmospheric Administration)^11^ or NASA (NASA Earth Observatory)^12^ for these fishing areas, which have been used in previous scientific explorations of this dataset^1^. As sea surface temperatures may not be as useful as bottom temperatures for benthic species, the latter could be obtained retrospectively from ocean models from websites such as HYCOM (Hybrid Coordinate Ocean Model)^13^.

Data are provided in a CSV format. Care must be taken with conversion of columns containing DD/MM/YYYY data from CSV files. Therefore, sampling year and sampling month are provided as separate fields.

## Data Availability

There was no custom R code produced during the collation and validation of this dataset.
